# Umbilical cord blood stem cells as third-party adjuvant infusions in human leukocyte antigen antibody-positive patients undergoing haploidentical hematopoietic stem cell transplantation

**DOI:** 10.3389/fimmu.2024.1459699

**Published:** 2024-09-27

**Authors:** Yuying Wang, Yiou Zhao, Xiaosheng Fang, Dai Yuan, Mei Ding, Kang Lu, Huiting Qu, Na Wang, Xiao Lv, Peipei Li, Changqing Zhen, Hongzhi Xu, Yujie Jiang

**Affiliations:** ^1^ Shandong Provincial Hospital Affiliated to Shandong First Medical University, Jinan, Shandong, China; ^2^ School of Life Science and Technology, Changchun University of Science and Technology, Changchun, Jilin, China; ^3^ Department of Hematology, Shandong Provincial Hospital Affiliated to Shandong First Medical University, Jinan, Shandong, China

**Keywords:** unrelated umbilical cord blood, haploidentical hematopoietic stem cell transplantation, graft failure, poor graft function, graft-versus-host disease, relapse-free survival

## Abstract

**Introduction:**

Graft failure (GF) or poor graft function (PGF) remain critical obstacles in haploidentical hematopoietic stem cell transplantation (haplo-HSCT), especially in recipients with HLA antibodies. Here, we performed a retrospective cohort study to investigate the efficacy and safety of the use of unrelated umbilical cord blood stem cells (UCBs) as a third-party adjuvant infusion in patients with HLA-antibodies undergoing haplo-HSCT.

**Methods:**

A total of 90 patients were divided into three groups: 17 patients in Group A (with positive HLA antibodies and who received UCB infusion), 36 patients in Group B (with positive HLA antibodies without UCB infusion), and 37 patients in Group C (without HLA antibody or UCB infusion).

**Results:**

The median age of patients included in Groups A, B, and C was 43 (IQR, 27 - 49.5), 33 (IQR, 20 - 48.75), and 30 (IQR, 18 - 46.5) years, respectively. All but one patient in Group B achieved granulocyte recovery within 28 days after transplantation. The median time to granulocyte engraftment were all 12 days for patients in Groups A, B, and C, respectively. All the patients in Group A achieved 100% donor chimerism without UCB engraftment. There were no significant differences in granulocyte or platelet engraftment time between the three groups. There were 1, 5, and 0 patients in Groups A, B, and C, respectively, who developed PGF. The cumulative incidence rates for any grade of acute graft-versus-host disease (aGVHD) were comparable among the three groups. Patients in Group B presented a greater incidence of cGVHD than did those in Group A (P = 0.002) and Group C (P = 0.006). Patients in Group A presented more limited and milder cGVHD than those in Group C (P < 0.0001). The 1-year relapse-free survival (RFS) was 70.6% (95% CI, 0.47 - 0.87), 55.6% (95% CI, 0.40 - 0.70), and 77.9% (95% CI, 0.63 - 0.89) in Groups A, B, and C, respectively.

**Discussion:**

Our results indicated that patients who were positive for HLA antibodies were at a greater risk of developing GF/PGF. Co-infusion with UCBs was safe and improved engraftment, cGVHD, and improved the 1-year RFS to some extent.

## 1 Introduction

With the progression of haploidentical hematopoietic stem cell transplantation (haplo-HSCT) technology, almost every patient with malignant hematopoietic diseases can find a donor receiving allogeneic-HSCT and achieve long overall survival (OS). However, the therapeutic benefits and wider application of haplo-HSCT are limited by graft-versus-host disease (GVHD), the latter remains a major obstacle to long-term survival for this population. Furthermore, rejection remains a critical reason for graft failure (GF) in the haplo-HSCT setting. The incidence of rejection was <3% for matched human leukocyte antigen (HLA)-identical sibling donors (MSDs) or matched unrelated donors (MUDs), and these data increased to >10% for haplo-HSCT. Furthermore, the incidence of poor graft function (PGF) with complete donor chimerism is also greater in the haplo-HSCT setting (1). Both GF and PGF often result in an increased incidence of transplant-related mortality (TRM) and inferior OS.

Previous studies have suggested that rejection is mainly related to donor-specific antibody (DSA), severe acute GVHD (aGVHD), HLA mismatching, stem cell number, etc. (2). DSA is the most important risk factor for rejection, and a previous study confirmed that DSA is the only risk factor for GF (3). In haploidentical donor selection, due to the presence of multiple donor-recipient mismatches, anti-HLA antibody screening must be performed in the recipient to detect the presence of DSA. In 2018, the European Society for Blood and Marrow Transplantation (EBMT) recommended DSA testing in all haploidentical donor transplant recipients and suggested an MFI > 1,000 as DSA positivity (4). If multiple donors are available, DSA-positive donors should be avoided. Furthermore, donors who have the same allele as patients with DSA (MFI ≥ 10,000) should be excluded. For patients with HLA antibodies but not DSAs, transplantation can be conducted as scheduled. However, these patients also have a higher GF rate than those without HLA antibodies, especially in haplo-HSCT (5). To date, there is no consensus on whether HLA antibodies but not DSAs should be managed before transplantation, and there is no ideal strategy for eradicating or decreasing HLA antibodies in this population (6).

Umbilical cord blood cells (UCBs) are characterized by abundant stem cell sources and low immunogenicity. UCB has a greater number of natural killer (NK) cells and a greater proportion of immature T cells (7). UCBs have previously been shown to contain a distinct regulatory T-cell (Treg) subset that exists at a relatively high frequency compared to that in peripheral blood (PB). Tregs and other components of UCB may act as immunomodulators to reduce immune rejection and regulate the hematopoietic microenvironment (7). Previous studies reported satisfactory results using UCBs as third-party stem cells in haplo- or MUD transplantations (8, 9). Lyu et al. confirmed a superior outcome of haplo-HSCT combined with third-party UCBs compared with MUD transplantation in 66 patients with a high risk of hematopoietic neoplasm relapse (10). Here, we hypothesized that third-party UCBs could offer some advantages for patients with HLA antibodies and decrease the incidence of GF/PGF.

In this retrospective, single-center, controlled study, we aimed to investigate the efficacy and safety of unrelated UCBs as third-party adjuvant infusions in patients with HLA antibodies receiving haplo-HSCT. The primary objectives were the incidence of GF/PGF and the transplant-related mortality (TRM) within 100 days posttransplantation. The secondary objectives included the following: incidence of aGVHD grades II-IV, chronic GVHD (cGVHD), relapse-free survival (RFS), GVHD- and relapse-free survival (GRFS), and overall survival (OS).

## 2 Material and methods

### 2.1 Patients and controls

**Figure 1:**
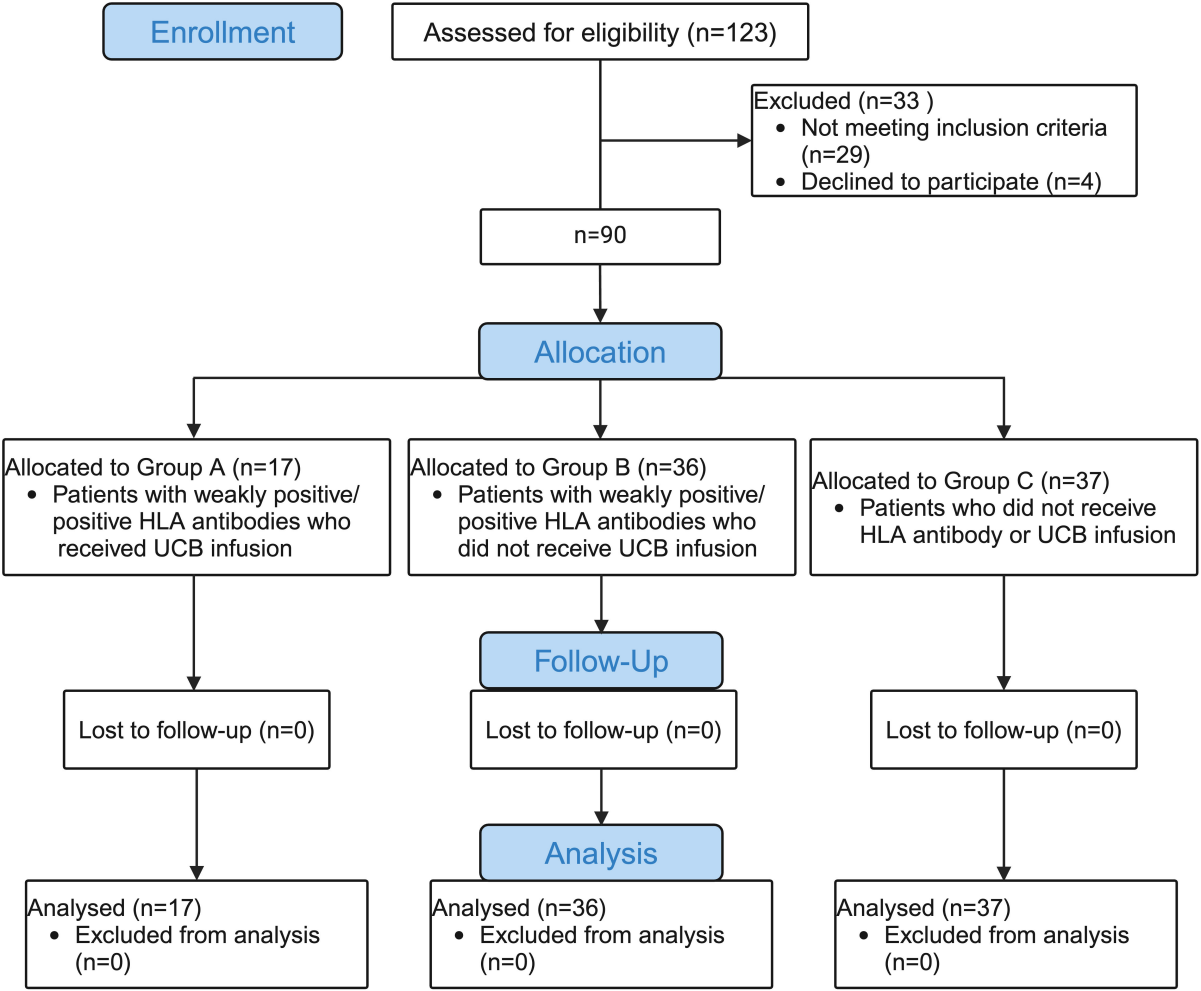
The consort flow chart of the study.

A total of 90 eligible participants with hematological diseases who underwent haplo-HSCT from May 2017 to December 2022 in the Department of Hematology of Shandong Provincial Hospital Affiliated to Shandong First Medical University were enrolled in this study. The inclusion criteria for patients were haplo-HSCT with weakly positive/positive HLA antibodies (MFI >500) but not DSAs. Age- and sex-matched patients who underwent haplo-HSCT without any HLA antibodies were chosen concurrently as controls. The exclusion criterion was haplo-HSCT with positive DSAs (MFI>1,000). HLA antibodies from each patient were routinely tested before transplantation. In this retrospective study, participants were assigned to three groups in a ratio of 1:2:2. Group A (experimental group: patients with weakly positive/positive HLA antibodies who received UCB infusion), Group B (positive control group: patients with weakly positive/positive HLA antibodies who did not receive UCB infusion), Group C (blank control group: patients who did not receive HLA antibody or UCB infusion). All protocols and consent forms were obtained from the patients or their guardians and approved by the Human Subjects Review Committee of the Shandong Provincial Hospital Affiliated to Shandong First Medical University. This project is registered by the Shandong Data Protection Agency and approved by the Joint Ethics Committee of Jinan. UCBs were obtained from the Shandong Umbilical Cord Blood Bank. All the study procedures were conducted in compliance with the Declaration of Helsinki. The consort flow chart of this study is shown in **Figure 1**.

### 2.2 Conditioning and prophylaxis for aGVHD

Patients included in this study received myeloablative conditioning (MAC), which included antithymocyte globulin (ATG) at a total of 10 mg/kg administered on days -5 to -2 before the transplant. The conditioning regimens used in this study are summarized in **Table 1**. The calcineurin inhibitor (CCI) combined with mycophenolate mofetil (MMF) and short-term methotrexate (MTX, 15 mg/kg on days +1, 10 mg/kg on days +3, +5, and +11) were administered for aGVHD prophylaxis. Glucocorticoid (GC)-based treatment was given when Grade II or higher aGVHD developed. For patients who developed steroid-resistant/refractory aGVHD (SR-aGVHD), second-line strategies, including ruxolitinib, anti-CD25 monoclonal antibody, and mesenchymal stem cell (MSC) infusion, were considered. For patients with cGVHD, GC combined with CCIs was the most common strategy used in our study.

**Table 1:** Demographic and clinical characteristics of patients and donors.

	No. (N = 90)	%
Donor		
Median age at mobilization, yr (IQR)	33.5(23.75 - 47)	–
Male	64	71.1%
Female	26	28.9%
Patient		
Median age at transplant, yr (IQR)	33(19 - 48)	–
Male	51	56.7%
Female	39	43.3%
Diagnosis		
AML, MDS	43	47.8%
ALL, MPAL	27	30.0%
CMML, CML	16	17.8%
SAA and other benign diseases	4	4.4%
Remission status at transplant		
CR1	59	65.6%
≥CR2	16	17.8%
NR	11	12.2%
SAA and other benign diseases	4	4.4%
Conditioning regimen		
IDA+BU/FLU	21	23.3%
BU/FLU	12	13.3%
BU/CY	6	6.7%
mBU/CY	4	4.4%
VP16+BU/CY	20	22.2%
TBI/CY	3	3.3%
FLU/CY	3	3.3%
Others	21	23.3%
Donor–patient gender matching		
Male-Female	28	31.1%
Male-Male	36	40.0%
Female-Male	15	16.7%
Female-Female	11	12.2%
Donor–patient relationship		
Patient-child	40	44.4%
Child-patient	34	37.8%
Sibling	16	17.8%
HLA match		
>5/10	15	16.7%
5/10	75	83.3%
Donor–patient blood type		
Match	62	68.9%
Mismatch	28	31.1%
Cellularity in haplo-grafts, median (IQR)		
Mononuclear cells, 10^8^/kg	10.46(8.16 - 13.26)	
CD34+ cells, 10^6^/kg	3.94(2.69 - 4.95)	

AML, acute myelocytic leukemia; MDS, myelodysplastic syndrome; ALL, acute lymphocytic leukemia; MPAL, mixed phenotype acute leukemia; CMML, chronic myelomonocytic leukemia; CML, chronic myelogenous leukemia; SAA, severe aplastic anemia; CR, complete remission; NR, non-remission; IDA, idarubicin; BU, busulfan; FLU, fludarabin; CY, cyclophosphamide; mBU/CY, modified BU/CY; VP16, etoposide; TBI, total body irradiation. IQR, interquartile range.

Acyclovir, fluconazole/posaconazole, and cotrimoxazole were administered prophylactically against herpes viruses, fungi, and pneumocystis pneumonia, respectively. Granulocyte colony-stimulating factor (G-CSF) was administered subcutaneously at a dose of 5 - 10 mg/kg from day +6 after stem cell transfusion and was discontinued until neutrophil engraftment. All blood products were irradiated, and leukocytes were depleted.

### 2.3 Transplant products

All patients received peripheral blood stem cells (PBSCs) from their haplo-donors. For patients in Group A, single unrelated UCBs were infused four hours before haplo-donor stem cells. The following criteria for cord blood unit selection were applied: 1) HLA matching was preferred at > 4/6 loci (HLA-A, HLA-B, and HLA-DRB1 loci serological matching) between donors and recipients. 2) The total nucleated cells (TNCs) were not less than 1 × 10^7^/kg of the recipient’s body weight after thawing. 3) Blood type-matched cord blood was preferred at an equal level of HLA-type matching. The matching landscape of the haplotype donor and cord blood is shown in **Table 2**.

**Table 2:** The cellularity of the stem cells and the engraftment time of neutrophil and platelet.

	Group A (n = 17)	Group B (n = 36)	Group C (n = 37)
HLA match (Cord blood)			
5/10	3 (17.6%)	–	–
>5/10	14 (82.4%)	–	–
Donor–patient blood type (Cord blood)			
Match	15 (88.2%)	–	–
Mismatch	2 (11.8%)	–	–
Cord blood TNC, 10^8^/kg			
Median (IQR)	15.16 (12.45 – 18.23)	–	–
Cord blood CD34+, 10^6^/kg			
Median (IQR)	4.63 (3.75 – 5.55)	–	–
Cell compositions in haplo-grafts, median (IQR)			
MNC, 10^8^/kg	11.03 (9.43 – 17.35)	9.95 (7.70 – 11.99)	10.80 (8.20 – 13.69)
CD34+ cells, 10^6^/kg	3.58 (2.58 – 4.81)	4.01 (2.61 – 6.50)	4.13 (2.85 – 4.87)
Granulocyte recovery at +28 days, n (%)			
Median (IQR)	12 (10.5 – 15)	12 (11 – 16)	12 (11 – 14)
Platelet recovery at +28 days, n (%)			
Median (IQR)	14 (12 – 16)	13 (12 – 17)	13 (12 – 17.75)

HLA, human leukocyte antigen; TNC, total nucleated cells; MNC, mononuclear cell; IQR, interquartile range.

### 2.4 Definitions and patient management

The first day of absolute neutrophil count (ANC) > 0.5 × 10^9^/L for 3 consecutive days was defined as neutrophil engraftment. The first day when the platelet count was > 20 × 10^9^/L without platelet transfusion support for 7 consecutive days was defined as platelet engraftment. Primary GF was defined as failure to achieve neutrophil engraftment within the first 28 days posttransplantation and lack of evidence of donor-type implantation. Poor graft function (PGF) was defined as the presence of multilineage cytopenias in the presence of 100% donor chimerism. The diagnostic and grading criteria for acute and chronic GVHD were determined according to the EBMT-NIH-CIBMTR Working Group Position Statement on Criteria and Guidelines for the Evaluation of Graft-versus-Host Disease (11). Serum levels of cytomegalovirus (CMV)- and Epstein‒Barr virus (EBV)-DNA were monitored twice weekly during the first 30 days posttransplantation and every month thereafter. Donor chimerism was evaluated every month in the first year and every 3 months until 3 years posttransplantation.

### 2.5 Data collection

The occurrence of granulocyte and platelet engraftment time, GVHD, CMV/EBV reactivation, hemorrhagic cystitis (HC), TRM within 100 days posttransplantation, 1-year relapse-free survival (RFS), 1-year GVHD- and relapse-free survival (GRFS), and 1-year overall survival (OS) between the three groups were analyzed in this study.

### 2.6 Statistical analysis

SPSS 26.0 software (SPSS, Chicago, IL) and GraphPad Prism software (La Jolla, CA) were used to conduct the statistical analyses and construct the figures. All quantitative values are expressed as the mean (range) or median (IQR, interquartile range). The normality of the continuous variables was assessed by the Kolmogorov-Smirnov test (K-S test). The Kruskal-Wallis test and analysis of variance (ANOVA) were used for comparisons among the three groups. A nonparametric test (Mann-Whitney U test) was used to compare the nonnormally distributed continuous variables between the two groups. The chi-squared test and Fisher’s exact probability test were performed to analyze categorical variables, including the incidence and grade of aGVHD, cGVHD, incidence of CMV, EBV reactivation, and HC, among the different groups. The TRM, RFS, GRFS, and OS were estimated using the Kaplan-Meier product limit estimation method, and differences in subgroups were assessed by the log-rank test. Throughout, two-sided P values < 0.05 were obtained via t-tests, and 95% confidence intervals (95% CIs) that included unity were considered to indicate statistical significance.

## 3 Results

### 3.1 Patient characteristics

A total of 90 patients were included in this study (17 in Group A, 36 in Group B, and 37 in Group C). The median age of patients included in Groups A, B, and C was 43 (IQR, 27 - 49.5), 33 (IQR, 20 - 48.75), and 30 (IQR, 18 - 46.5) years, respectively. These participants were aged 8 to 65 years, and the median age of the patients at transplantation was 33 years. The clinical characteristics of the patients and the paired donors are listed in **Table 1**. The median follow-up time was 26.5 (range, 1 - 79 months), and the last follow-up time point was December 2023.

### 3.2 HLA antibody distribution

**Figure 2:**
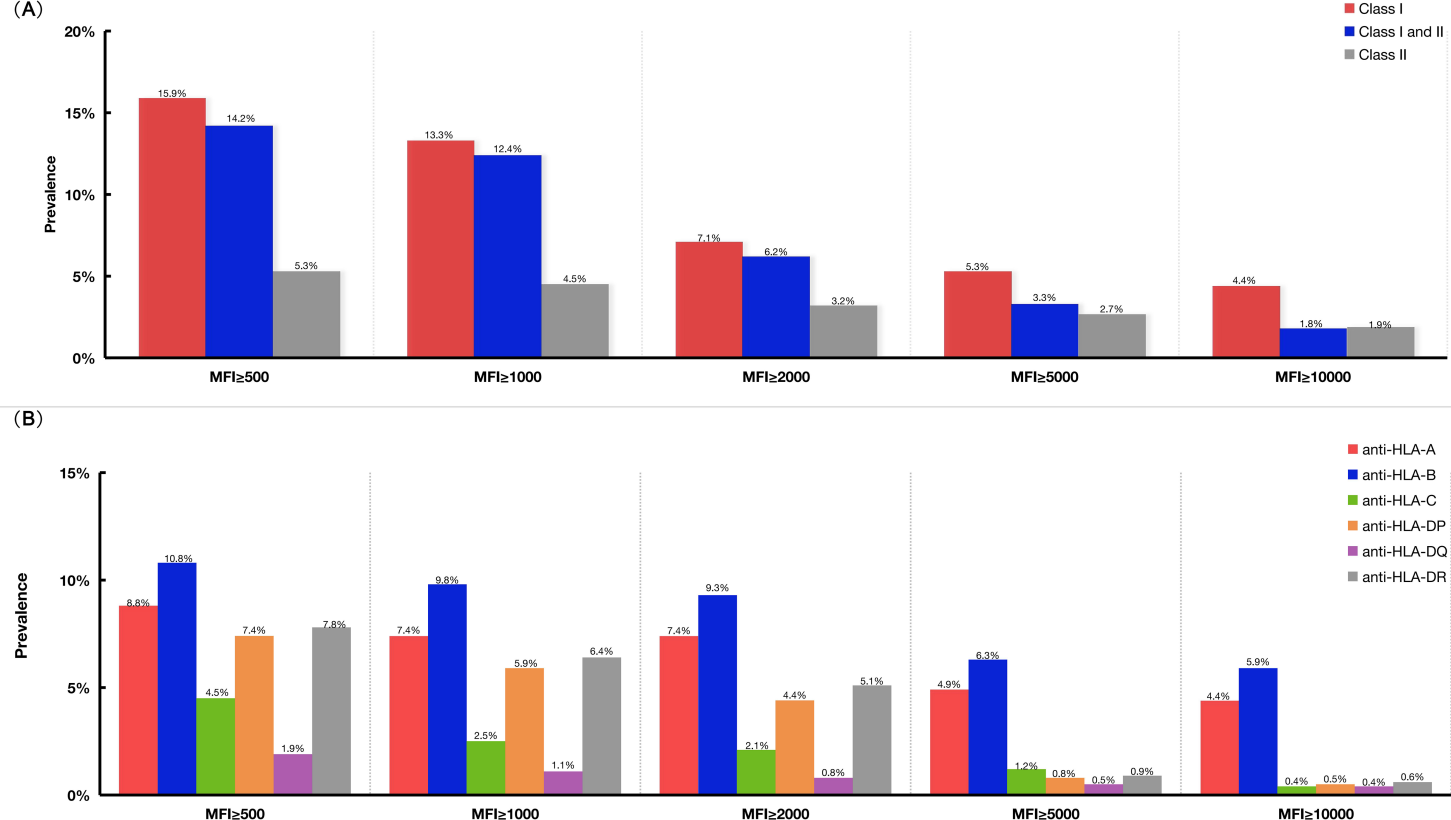
The prevalence of HLA antibodies according to different cut-off values of MFI in Groups A and B. **(A)** The prevalence of HLA antibodies in Group A and Group B patients according to different MFI cut-off values. **(B)** The prevalence of HLA antibodies specific for antigens coded by different HLA loci, such as HLA-A, -B, -C, -DP, -DQ, -DR. HLA, human leukocyte antigen; MFI, mean fluorescence intensity.

The prevalence of antibodies to HLA according to different cutoff values of mean fluorescence intensity in Groups A and B are shown in **Figures 2A, B**. For patients with HLA antibodies, the three most frequent alleles were HLA-B76, HLA-DP1, and HLA-DR4. The first three highest MFI values were 23,768, 22,741, and 22,428, which corresponded to the HLA loci HLA-B13, HLA-B61, and HLA-B60, respectively. One patient in Group B who developed GF presented with a weakly positive HLA-A02 (MFI 1294) antibody.

### 3.3 Transplant products and engraftment

For all 90 participants, the median numbers of reinfused haplo-graft mononuclear cells (MNCs) and CD34+ cells were 10.46 (IQR, 8.16 - 13.26) × 10^8^/kg and 3.94 (IQR, 2.69 - 4.95) × 10^6^/kg, respectively. The median MNCs and CD34+ cells for the three groups were compared, and there was no significant difference among them.

No obvious adverse effects were observed during the process of UCB infusion. For Group A, the median cellularity of the third-party UCB units was 15.16 (IQR, 12.45 - 18.23) × 10^8^/kg for TNCs and 4.63 (IQR, 3.75 - 5.55) × 10^6^/kg for CD34+ cells. The median number of MNCs in the UCBs was 11.03 (IQR, 9.43 - 17.35) × 10^8^/kg, and the median number of CD34+ cells was 3.58 (IQR, 2.58 - 4.81) × 10^6^/kg.

All the patients in Group A achieved 100% donor chimerism without UCB engraftment. There were no significant differences in granulocyte or platelet engraftment time between Group B and the other two groups.

The median time to granulocyte engraftment was 12 (IQR, 10.5 - 15), 12 (IQR, 11 - 16), and 12 (IQR, 11 - 14) days for patients in Groups A, B, and C, respectively. The median time to platelet recovery was 14 (IQR, 12 - 16), 13 (IQR, 12 - 17), and 13 (IQR, 12 - 17.75) days in Groups A, B, and C, respectively. The cellularity of the stem cells and the graft times of the granulocytes and platelets in the three groups are shown in **Table 2**.

### 3.4 Incidence of GF or PGF

All but one patient in Group B achieved granulocyte recovery within 28 days after transplantation. This patient died of multiple organ dysfunction syndrome (MODS) at 50 days posttransplantation.

There were 1, 5, and 0 patients in Groups A, B, and C, respectively, who developed PGF. In Group A, the patient who developed PGF received a child-parent donation with a matched blood type. The HLA-antibody MFI of this patient was 18,649.3, which corresponded to the HLA locus of HLA-B13. The number of MNCs and CD34+ cells from his haplo-donor group were 11.89 × 10^8^/kg and 4.7 × 10^6^/kg, respectively. This patient eventually died from a serious infection. According to Group B, 4 males and 1 female developed PGF. There were 3 parent-child and 2 child-parent donor-patient relationships. Three donor-recipient blood types were matched, and 2 donor-recipient blood types were not matched. The mean numbers of MNCs and CD34+ cells in these five patients were 9.90 (range, 7.01 - 15.2) × 10^8^/kg and 4.75 (range, 2.33 - 8.52) × 10^6^/kg, respectively. All five patients died, two died of severe infection, two died of respiratory and circulatory failure, and one died of posttransplant lymphoproliferative disease (PTLD).

### 3.5 The incidence of aGVHD and cGVHD

**Figure 3:**
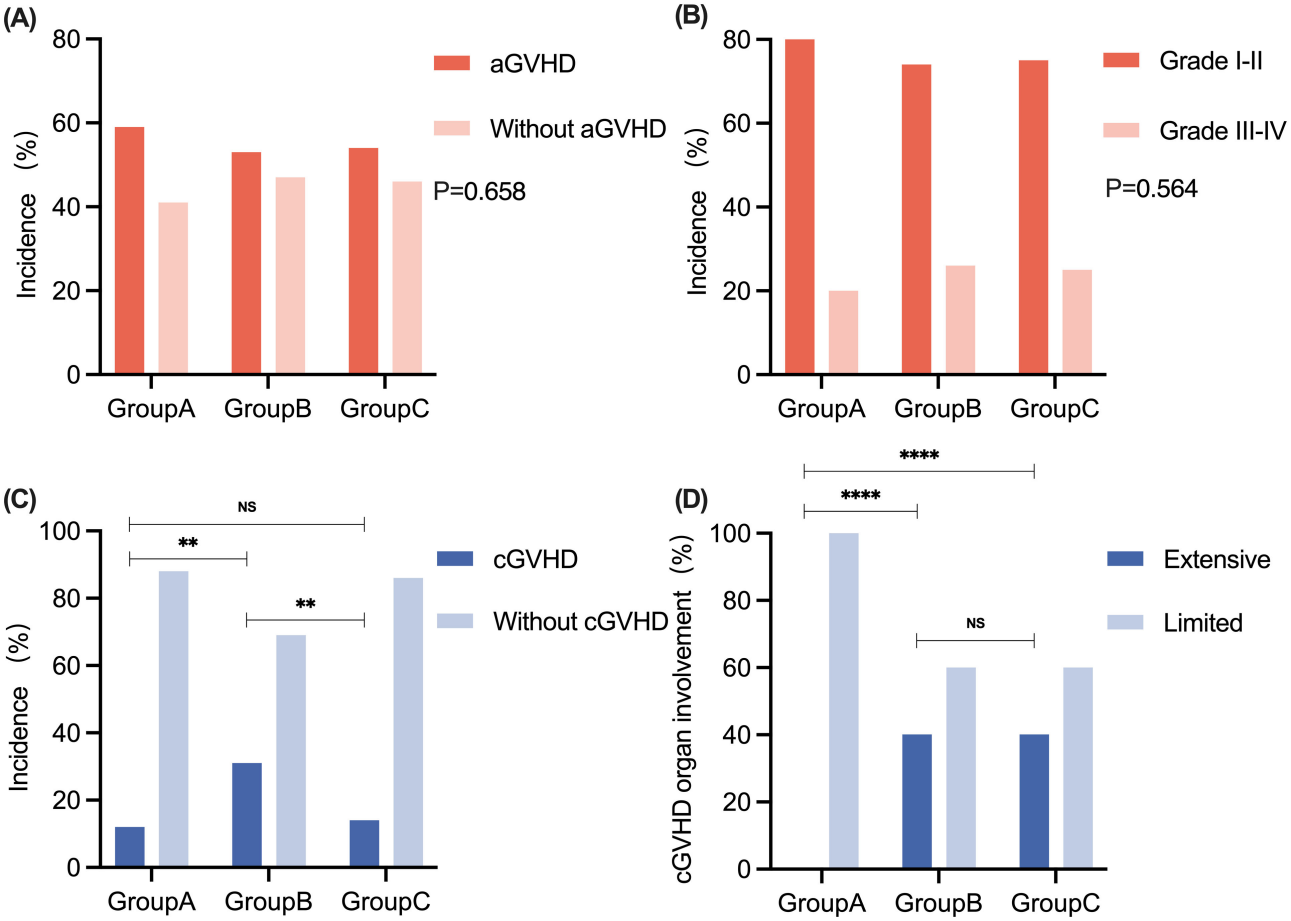
The comparison of aGVHD and cGVHD among patients in Groups A, B, and C. **(A)** The incidence of aGVHD in Groups A, B, and C. **(B)** The grades of aGVHD in Groups A, B, and C. **(C)** The incidence of cGVHD in Groups A, B, and C. **(D)** The degree of cGVHD organ involvement in Groups A, B, and C. No significant difference was observed in the incidence and grading of aGVHD between patients in Groups A, B, and C, respectively. **P < 0.01. ****P < 0.0001. aGVHD, acute graft-versus-host disease; cGVHD, chronic graft-versus-host disease; NS, no significance.

In all 90 patients, the cumulative incidence rates for Grade I-II aGVHD and Grade III-IV aGVHD were 75.5% (37/90) and 24.5% (12/90), respectively. Eight patients (1 patient in Group A, 3 patients in Group B, and 4 patients in Group C) developed grade IV intestinal aGVHD, three of whom developed SR-aGVHD, and all of whom responded to second-line anti-aGVHD therapy. There were no significant differences in the incidence or grades of aGVHD among the patients in the three groups (**Figures 3A, B**). The median time points of aGVHD occurrence were 17.5 (range, 8 - 31), 21 (range, 10 - 45), and 20 (range, 11 - 31) days after transplant for Groups A, B, and C, respectively. The clinical characteristics and grades of aGVHD in the three groups of patients are shown in **Table 3**. The incidence of cGVHD in 84 patients who survived to +100 days was evaluated. The cumulative incidence rate of cGVHD was 20.2% (17/84). There were 2, 10, and 5 patients who developed cGVHD in Groups A, B, and C, respectively. There was no significant difference in the incidence of cGVHD between Group A and Group C. However, patients in Group B presented a greater incidence of cGVHD than did patients in the other two groups (Group B vs. Group A: 31.3% vs. 12.5%, P = 0.002; Group B vs. Group C: 31.3% vs. 13.9%, P = 0.006) (**Figure 3C**). Patients in Group A presented with limited cGVHD, which was relatively mild compared with that in Group B and Group C (0% vs. 40%, P < 0.0001, **Figure 3D**). There was no statistically significant difference in the degree of cGVHD organ involvement between patients in Group B and those in Group C.

**Table 3:** The clinical characteristics of patients with aGVHD/cGVHD.

	Group A	Group B	Group C	P-value
**Occurrence of aGVHD, n (%)**	10 (58.8)	19 (52.8)	20 (54.1)	0.658
**Median time of aGVHD occurrence, days (range)**	17.5 (8 – 31)	21 (10 – 45)	20 (11 – 31)	0.553
**Mean age, yr (range)**	37.60 (16 – 63)	29.42 (9 – 65)	30.95 (14 – 56)	0.110
**Gender**				0.222
Male, n (%)	6 (60.0)	10 (52.6)	13 (65.0)	–
Female, n (%)	4 (40.0)	9 (47.4)	7 (35.0)	–
**Primary disease, n (%)**				0.482
AML, MDS	5 (50.0)	8 (42.1)	8 (40.0)	–
ALL, MPAL	3 (30.0)	6 (31.6)	9 (45.0)	–
CMML, CML	1 (10.0)	3 (15.8)	2 (10.0)	–
SAA and other benign diseases	1 (10.0)	2 (10.5)	1 (5.0)	–
**Pre-transplantation status, n (%)**				0.240
CR1	7 (70.0)	11 (57.9)	12 (60.0)	–
≥CR2	3 (30.0)	2 (10.5)	5 (25.0)	–
NR or other	0 (100.0)	6 (31.6)	3 (15.0)	–
**Grades of aGVHD, n (%)**				0.564
I	3 (30.0)	7 (36.8)	7 (35.0)	–
II	5 (50.0)	7 (36.8)	8 (40.0)	–
III-IV	2 (20.0)	5 (26.3)	5 (25.0)	–
**aGVHD organ involvement, n (%)**				0.051
Skin	6 (60.0)	9 (47.4)	8 (40.0)	–
Liver	1 (10.0)	0 (0.0)	0 (0.0)	–
Gastrointestinal tract	1 (10.0)	3 (15.8)	4 (20.0)	–
Two or more organs involved	2 (20.0)	7 (36.8)	8 (40.0)	–
**Occurrence of cGVHD, n (%)**	2 (12.5)	10 (31.3)	5 (13.9)	<0.001
**Median time of cGVHD occurrence, months (range)**	7 (5 – 9)	7 (3 – 23)	8 (3 – 12)	0.830
**Early aGVHD before cGVHD, n (%)**				0.048
Yes	2 (100.0)	5 (50.0)	3 (60.0)	–
No	0 (0.0)	5 (50.0)	2 (40.0)	–
**cGVHD Organ Involvement, n (%)**				<0.0001
Lung	0 (0.0)	1 (10.0)	0 (0.0)	–
Skin, joint, and connective tissue	0 (0.0)	0 (0.0)	2 (40.0)	–
Liver	0 (0.0)	3 (30.0)	1 (20.0)	–
Oral cavity	2 (100.0)	2 (20.0)	0 (0.0)	–
Two or more organs involved	0 (0.0)	4 (40.0)	2 (40.0)	–

AML, acute myelocytic leukemia; MDS, myelodysplastic syndrome; ALL, acute lymphocytic leukemia; MPAL, mixed phenotype acute leukemia; CMML, chronic myelomonocytic leukemia; CML, chronic myelogenous leukemia; SAA, severe aplastic anemia; CR, complete remission; NR, non-remission; aGVHD, acute graft-versus-host disease; cGVHD, chronic graft-versus-host disease.

At the end of follow-up, the median cGVHD durations of the three groups were 7 (range, 5 - 9), 7 (range, 3 - 23), and 8 (range, 3 - 12) months, respectively. The distribution of characteristics of patients with cGVHD in the three groups is shown in **Table 3**. The organs most frequently involved in cGVHD were the oral cavity (4/17, 23.5%) and liver (4/17, 23.5%). One patient in Group B developed bronchiolitis obliterans syndrome (BOS) and lived with poor quality of life at the last follow-up (**Supplementary Table S1**).

### 3.6 The incidence of CMV and EBV reactivation

**Figure 4:**
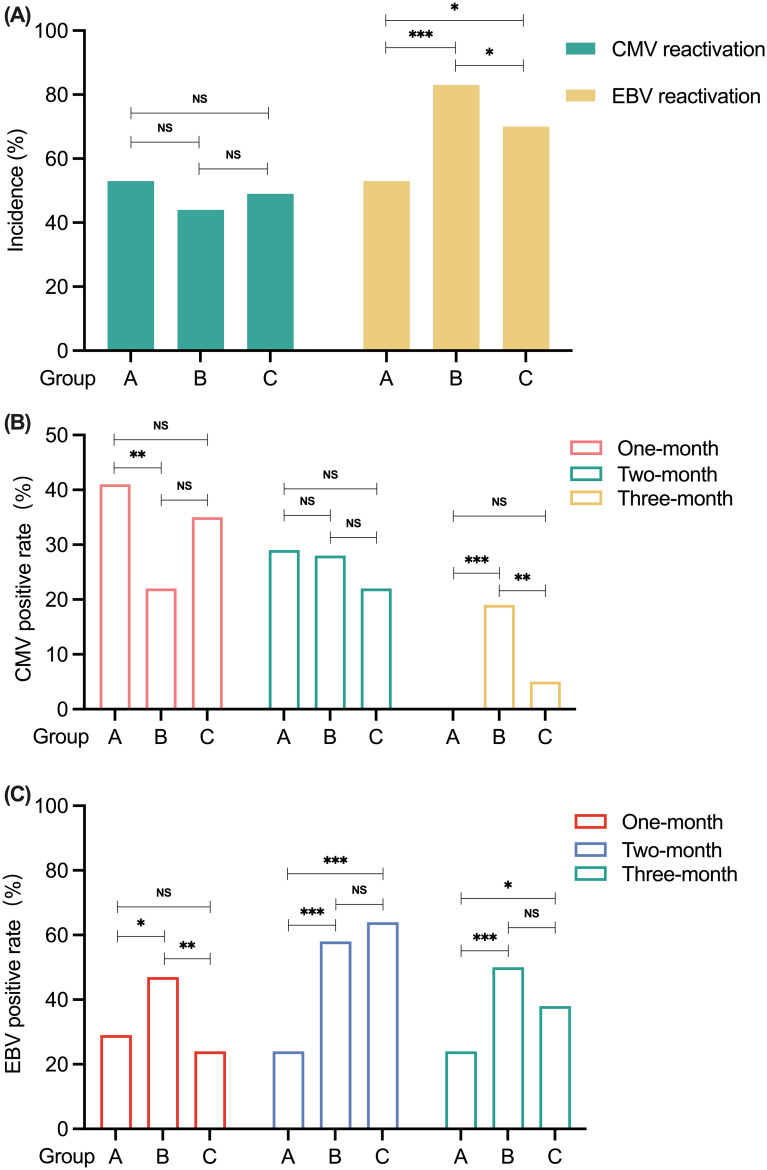
The comparison of CMV and EBV reactivation among patients in Groups A, B, and C. **(A)** The incidence of CMV and EBV reactivation within 100 days after transplantation in Groups A, B and C. **(B)** CMV reactivation in Groups A, B, and C by month post-transplantation. **(C)** EBV reactivation in Groups A, B, and C by month post-transplantation. *P < 0.05. **P < 0.01. ***P < 0.001. NS, no significance. CMV, cytomegalovirus; EBV, Epstein-Barr virus.

The differences in the reactivation of CMV and EBV among the three groups are presented in **Table 4**. The total incidences of CMV and EBV viremia were 47.8% (43/90) and 72.2% (65/90), respectively. As shown in **Figure 4A**, there were no significant differences in CMV reactivation among the three groups in the first 100 days after transplantation. In terms of EBV reactivation, patients in Group A presented with less EBV reactivation than did those in Group B (52.9% vs. 70.3%, P = 0.020) and Group C (52.9% vs. 83.3%, P < 0.001). Patients in Group B had a greater incidence of EBV reactivation than did those in Group C (83.3% vs. 70.3%, P = 0.045) in the first 100 days posttransplantation (**Figure 4A**). As shown in **Figure 4B**, when the data were analyzed by month, the rate of CMV reactivation was greater in Group A than in Group B at 1 month after transplantation (41.2% vs. 22.2%, P = 0.006), and there was no significant difference between Group C and any of the other two groups. There was no statistically significant difference in the percentage of patients who experienced CMV reactivation in the 2nd month after transplantation among the groups. At 3 months posttransplantation, no CMV reactivation occurred in Group A patients, and the rate of CMV reactivation was greater in Group B patients than in the other two groups (Group B vs. Group A 19.4% vs. 0.0%, P < 0.001; Group B vs. Group C 19.4% vs. 5.4%, P = 0.004).

**Table 4:** The reactivation of CMV and EBV between the three groups.

	Group A	Group B	Group C	P-value
CMV reactivation within 100 days				0.443
Yes	9(52.9)	16(44.4)	18(48.6)	–
No	8(47.1)	20(55.6)	19(51.4)	–
CMV reactivation by month				
1^st^-month	7(41.2)	8(22.2)	13(35.1)	0.014
2^nd^-month	5(29.4)	10(27.8)	8(21.6)	0.478
3^rd^-month	0(0.0)	7(19.4)	2(5.4)	<0.0001
EBV reactivation within 100 days				<0.0001
Yes	9(52.9)	30(83.3)	26(70.3)	–
No	8(47.1)	6(16.7)	11(29.7)	–
EBV reactivation by month				
1^st^-month	5(29.4)	17(47.2)	9(24.3)	0.001
2^nd^-month	4(23.5)	21(58.3)	23(63.9)	<0.0001
3^rd^-month	4(23.5)	18(50.0)	14(37.8)	<0.001

CMV, cytomegalovirus; EBV, Epstein-Barr virus.

When we analyzed the data by month, there was no significant difference in the rate of EBV reactivation between patients in Group A and those in Group C at 1 month posttransplantation (**Figure 4C**). However, the rate of EBV reactivation in Group B patients was greater than that in Groups A (47.2% vs. 29.4%, P = 0.013) and C (47.2% vs. 24.3%, P = 0.001). At 2 months posttransplantation, the rate of EBV reactivation in Group A patients was lower than that in Group B patients (23.5% vs. 58.3%, P < 0.001) and Group C patients (23.5% vs. 62.2%, P < 0.001). However, there was no significant difference between Groups B and C. At 3 months after transplantation, the percentage of patients who were positive for EBV reactivation was lower in Group A than in Group B (23.5% vs. 50.0%, P < 0.001) and Group C (23.5% vs. 37.8%, P = 0.046). Similarly, there was no significant difference in the rate of EBV reactivation between patients in Groups B and C.

In addition, a total of 4 patients developed PTLD posttransplantation (1 in Group A, 2 in Group B, and 1 in Group C). The patient in Group C has survived to date, and the patient in Group A died of hemophagocytic lymphohistiocytosis (HLH). Two patients in Group B died of PTLD complicated with severe infection secondary to hematopoietic failure.

### 3.7 Other complications posttransplantation

The mean incidence of HC in the first 100 days post-transplantation in Groups A, B, and C transplantation was 29.4% (5/17), 30.6% (11/36), and 27.03% (10/37), respectively. There was no significant difference in HC incidence among the three groups (all P values > 0.05).

Forty-three (47.8%) patients developed lung infections, and 38 (42.2%) patients suffered multiple organ infections (abdominal, sinus infections, etc.). There were no significant differences in the number of infectious organs or pathogens among the three groups.

### 3.8 Survival analysis

**Figure 5:**
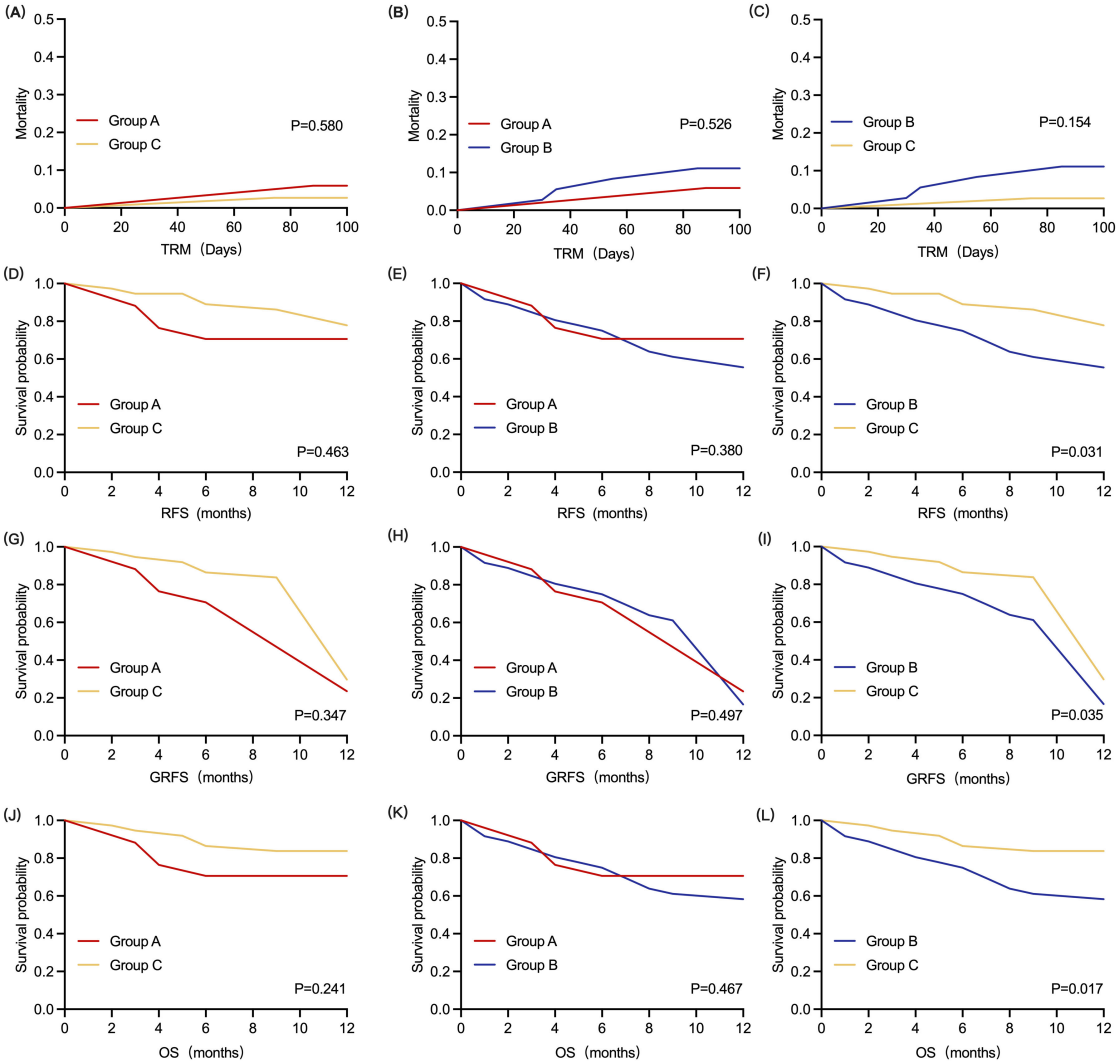
Kaplan-Meier analysis of all patients (Groups A, B, and C). **(A–C)** TRM within 100 days for all patients. **(D–F)** 1-year RFS for all patients. **(G–I)** 1-year GRFS for all patients. **(J–L)** 1-year OS for all patients. Significant differences in 1-year RFS, 1-year GRFS and 1-year OS were observed between patients in Group B and Group C, respectively (P < 0.05, log-rank test, for each cohort). TRM, transplant-related mortality; RFS, relapse-free survival; GRFS, GVHD- and relapse-free survival; OS, overall survival.

The TRM within 100 days after transplantation was 5.9%, 11.1%, and 2.7% in Groups A, B, and C, respectively. The comparison of TRM within 100 days after transplantation did not significantly differ among the three groups (**Figures 5A–C**).

The 1-year RFS rates were 70.6%, 55.6%, and 77.9% in Groups A, B, and C, respectively. The 1-year RFS of patients in Group A was not significantly different from that of patients in the other two groups (**Figures 5D, E**). However, the 1-year RFS of patients in Group C was greater than that of patients in Group B [HR = 0.41 (0.18 - 0.92), P = 0.031] (**Figure 5F**).

The 1-year GRFS for the three groups were 23.5%, 16.7%, and 29.7%, respectively. As shown in **Figures 5G** and **5H**. Compared with Groups B and C, the 1-year GRFS was not statistically significant in patients in Group A. The 1-year GFRS was higher in patients in Group C than in Group B [HR = 0.66 (0.39 - 1.12), P = 0.035] (**Figure 5I**).

After a median follow-up period of 26.5 (range, 1 - 79) months, 61 patients survived. The 1-year OS rates of patients in Groups A, B, and C were 70.6%, 58.3%, and 83.8%, respectively. As shown in **Figures 5J** and **5K**, there was no significant difference in the 1-year OS between patients in Group A and those in the other two groups. However, the 1-year OS of patients in Group C was greater than that of patients in Group B [HR = 0.35 (0.15 - 0.83, P = 0.017] (**Figure 5L**).

## 4 Discussion

In the present study, we investigated the efficacy and safety of unrelated UCBs as third-party adjuvant infusions in patients with HLA antibodies receiving haplo-HSCT. Preexisting or *de novo* HLA antibodies can be derived from blood transfusion, pregnancy, history of allogeneic transplantation, self-peptides, tumor antigens, CMV, influenza virus infection, bacterial infection, etc. (12, 13) There has been no consensus on the necessary and ideal strategy for eradicating or decreasing HLA antibodies before transplantation. However, former studies indicated that patients with HLA antibodies but not DSA are also at a higher risk of developing GF/PGF.

As alternative stem cells, UCBs are characterized by abundant stem cell sources and low immunogenicity (14, 15). UCBs are easy to achieve and have a high matching success rate. Lyu et al. confirmed that haplo-HSCT co-infused with third-party cord blood induced immune tolerance and modulated allogeneic reactions in patients who underwent mismatched HSCT (10). The incidence of grade II-IV aGVHD in their cohort was 14.3%, which was lower than that in previous reports of 28% (16). Cheng et al. confirmed a similar outcome with MSD-PBSCT when they infused cord blood stem cells with PBSCT in haplo-setting (8). The feasibility and efficacy of UCBs as third-party adjuvant infusions in previous studies may be related to low immunogenicity and immunomodulation. Therefore, we hypothesized that third-party UCBs could offer some advantages for patients with HLA antibodies at a high risk of GF/PGF.

In the present study, the incidence of GF/PGF in Group B (5 cases) was greater than that in Group A (1 case) and Group C (0 case), respectively, indicating that UCB transfusion could benefit the engraftment of haplo-donor stem cells in patients with HLA antibodies. The potential reasons for this improvement might include the following aspects. First, compared with BM/PB cells, UCB cells might have more robust differentiation and proliferation capabilities. Cairo et al. demonstrated an 80-fold increase in stem cell factor (SCF) and G-CSF or GM-CSF after a 14-day expansion of UCB versus adult BM using IL-11 (17). Moreover, compared with adult BM, UCB has been shown to have increased serial *in vitro* replating efficiency (18) and increased culture lifespan with increased progenitor cell production (19, 20). Second, the phenotype and constitution of the precursor cells in UCB are different from those in BM/PB cells. Hao et al. found that the primitive cell population that expresses the CD34 antigen is fourfold more prevalent than in BM or peripheral blood progenitor cells (PBPCs). This subpopulation of UCB has a greater *in vitro* cloning efficiency than the same population isolated from adult BM (21). Third, other components, such as Thy-1 antigen (CD90), expressed on UCB progenitors might have synergistic effects on precursor cell differentiation (22). Thy-1 is thought to assist in hematopoietic cell development. These data support that UCB has more primitive immature colony-forming cells (CFCs) than adult BM/PB and that these hematopoietic progenitors are capable of long-term repopulation (23). Last, it has been shown that stimulated NKT cells can facilitate hematopoietic reconstitution through dual immunoregulatory functions by secreting IL-10, IL-4, and TGF-β through the direct cell-cell contact pathway or cytokine pathway to induce immune tolerance and improve immune survival (7). Overall, due to their robust differentiation and proliferation capabilities, different phenotypes and constitutions of precursor cells, and ability to stimulate NKT cells, UCBs might facilitate haplo-donor stem cell homing and differentiation after transplantation, thus overcoming the adverse effects of HLA-antibodies on engraftment.

In our study, the cumulative incidence rates for grades I-II aGVHD, grades III–IV aGVHD, and cGVHD were 41.1% (37/90), 13.3% (12/90), and 18.9% (17/90) respectively, which is similar to that in previously reported studies (16, 24, 25).

We also observed a slight increase in Grade IV intestinal aGVHD in Groups B (3 patients) and C (4 patients) compared with Group A (1 patient). The exact mechanism by which UCBs improve aGVHD efficacy is not yet fully understood. The reasons may include the following aspects. First, in contrast to the complexity of T cells in the PB, T cells are largely naïve in UCB (26). Tregs are a subset of CD4+ T cells that are known to limit inflammatory reactions, and they could be considered for prophylaxis and treatment of severe and refractory GVHD (27). UCB contains a significant number of CD4+CD25+ Tregs and mesenchymal stem cells (MSCs), which have immune regulatory mechanisms that can regulate the hematopoietic microenvironment and therefore play important roles in the prevention of GVHD. Clinical-grade expansion of Tregs from UCB has been performed successfully (28) *in vitro*. Second, UCB is a rich source of NK cells. Janelle A Olson et al. investigated the impact of donor NK cells on donor alloreactive T cells in GVHD induction in an animal model. Donor T cells exhibited less proliferation, lower CD25 expression, and decreased interferon-gamma (IFN-gamma) production in the presence of donor NK cells. Interestingly, the GVL effect was retained in the presence of donor NK cells (29). Third, type II NKT cells can secrete IL-4 to induce Th2-type immune responses, both of which together contribute to alleviating GVHD (30). Pillai et al. reported that CD4-CD8-NKT cells, a subtype of NKT cells, were able to kill T cells and APCs expressing CD1d molecules, thereby inhibiting immune cell proliferation recognized by specific antigens and alleviating aGVHD (31). Larger samples are needed to validate the efficacy of UCB co-infusion in mitigating aGVHD.

In the present study, it is worth noting that patients in Group B presented a greater incidence of cGVHD than did patients in the other two groups. Patients in Group A presented more limited cGVHD than did patients in Group C. The pathophysiology of cGVHD is characterized mainly by impaired immune tolerance, and alloreactive donor-derived T and B cells are involved in this process (32). During cGVHD, regulatory cell populations, including Tregs, regulatory B cells (Bregs), regulatory NK cells, invariant NKT cells, and regulatory type 1 T cells, are impaired or reduced in frequency or number, resulting in the continuous release of inflammatory factors and ultimately pathogenic immunoglobulin deposition in various organs (33, 34). The history and severity of aGVHD are the strongest predictors among the known risk factors for cGVHD (26). The abundance of Tregs, Bregs, and NK cells in UCB might benefit the immune reconstitution, and alleviate aGVHD, thus improving cGVHD in patients who received a co-infusion of UCBs. In the future, more investigations performed both *in vivo* and *in vitro* are needed to confirm our findings.

In the present study, we did not find a significant difference in the TRM within 100 days after transplantation among the three groups. The 1-year RFS and 1-year GRFS of patients in Group A were not significantly different from those in the other two groups. However, if patients with HLA-antibodies did not receive UCB transfusion, their 1-year RFS was obviously lower than that of patients in Group C. These findings indicated that the presence of HLA antibodies was an adverse factor for relapse and that the co-infusion of UCBs can reverse this adverse effect to some extent. Yang et al. confirmed that the addition of UCBs could improve the prognosis of patients receiving haplo-HSCT and enhance the GVL effect without increasing the incidence of GVHD (35). This might be interpreted that faster engraftment and immune reconstitution in Group A may contribute to a GVL effect. In our previous perception, recipients who develop PGF will be at a greater risk of relapse than recipients who achieve good hematopoietic reconstitution. The GVL effect is considerably dependent on the amount and function of NK cells, especially allogenic mismatched NK cells. The effects of GVL can also be initiated by antigen-specific T cells and activated dendritic cells (DCs) of leukemic origin (36). The concurrent increase in NKT cell numbers and activities, the promoted host DC activation, subsequent CD8-dependent GVL effects, and increased generation of Tregs can all contribute to the preservation of the GVL and the prevention of CD4-dependent GVHD. Compared with NK cells derived from PB, cord blood-derived NK cells are younger, proliferate more, and have greater efficacy in targeting and killing malignant cells (19). Many NK progenitor cell populations can be found in UCB, and these populations are usually not present in PB (37). These include the CD34-CD133-CD7-CD45+lin- population, which can differentiate into NK cells after culture with IL-15 and stromal cells. Furthermore, CD34+CD7+ and CD34-CD7+ progenitor cells were also more abundant in UCB, and these cells were also able to develop into NK cells (13). Furthermore, when aGVHD is initiated, excessive cytokines can activate CTLs and NK cells and directly exert an immune response. Combined with our experimental results, co-infusion with UCBs in a haplo-HSCT setting might utilize this ‘shortcoming’ of increased risk of aGVHD and favor NK cells to facilitate the GVL effect. Therefore, the co-infusion of UCBs with haplo-stem cells might provide a certain number of progenitor cells and derived NK cells, and the latter will play an important role in anti-infection and anti-leukemia effects.Furthermore, there was no significant difference in the 1-year OS of patients in Group A compared with the other two groups. A larger sample size and longer follow-up are anticipated to draw more exact conclusions about the long-term survival of the participants in this study. Overall, co-infusion of UCBs might alleviate the relapse rate to some extent by potentially maintaining the GVL effect. The abundant NK cells and the cross-talk effect of cytokines might be involved in this complex process.

Our results indicated that the total incidences of CMV and EBV viremia were 47.8% and 72.2%, respectively, which is consistent with previous studies in developing countries. There were no significant differences in CMV reactivation among the three groups in the first 100 days after transplantation. Patients in Group A presented with less EBV reactivation than did those in Group B and Group C in the first 100 days posttransplantation. This may be due to the faster neutrophil reconstitution in Group A than in Group B. Furthermore, NK cells derived from UCBs might also be an important cause of less virus activation. NKT cells can activate the immune system involved in resisting viral and bacterial infection (38). The anti-infective effect of NKT cells is achieved by direct recognition of CD1d-presenting bacterially derived lipid antigens or by the development of responses to self-lipid antigens presented by APCs and infectious agents when interacting with pathogen-associated molecules (29). Therefore, the anti-infection ability of NKT cells can be used to effectively reduce the infection rate, improve the survival rate, and solve the problem of viral and fungal infection in the first few months after HSCT. Kotsianidis et al. (39) reported that NKT cells secrete hematopoietic-related cytokines, such as granulocyte giant cell colony-stimulating factor (GM-CSF) and IL-3, which are involved in regulating stable hematopoietic processes through CD1d recognition of hematopoietic precursor cells.

In addition, a total of 4 patients developed PTLD posttransplantation (1 in Group A, 2 in Group B, and 1 in Group C). EBV activation is the most important risk factor for developing PTLD. Patients in Group A might benefit from a reduced incidence of EBV activation due to cotransfusion with UCBs. In the future, a larger sample size should be investigated to determine why patients in Group A presented lower EBV activation than those in Group C.

In our study, a 4/6 match for HLA markers was the minimum criterion for choosing a UCB. As recommended by the EBMT, the minimum amount for UCB transplantation after thawing is 2.0 - 2.5 × 10^7^/kg for TNC and 1.0 - 1.2 × 10^5^/kg for CD34+ cells (40). Koen van Besien et al. proposed that a cell dose of > 3 × 10^6^ total cord blood units is the minimum threshold cell dose for haplo-cord engraftment. They also determined that a dose lower than 1 × 10^7^/kg has a high risk of rejection of the UCB graft (41). In our study, the median doses of third-party UCBs to the TNC and CD34+ cells were 15.16 × 10^7^/kg and 4.63 × 10^5^/kg, respectively. All the patients who achieved successful engraftment had haploid-stem cell engraftment without cord blood engraftment, which may be related to factors such as the number of haploid-stem cells being far greater than the number of cord blood hematopoietic stem cells. Furthermore, there might be both intrinsic cord blood factors and recipient factors that can lead to cord blood not engrafting. The exact mechanisms underlying the lack of UCB engraftment should be investigated in more in-depth studies. Our results indicated that the use of UCBs as a complement to haplo-HSCT is a feasible and safe strategy without concern about UCB engraftment.

Notably, our study has several limitations. First, the sample size was small, and the observation time was short. The clinical outcomes relating to survival and disease control seem to be preliminary. It would be better to classify the patients into subgroups according to their titers of HLA antibodies and analyze the differences between these groups. Second, as a retrospective cohort study, certain imbalanced features may exist in our study such as the unbalance in the number of cases between groups, although we made adjustments in the multivariate analysis. Finally, it is necessary to monitor HLA antibody titers after transplantation to find more evidence of the benefit of UCB.

## 5 Conclusions

In the present study, UCBs administered to patients who were positive for HLA antibodies as a third-party adjunctive infusion were safe and improved the engraftment, the grade and number of organs involved in cGVHD, decreased the incidence of EBV reactivation, and improved the 1-year RFS to some extent. Patients who are positive for HLA antibodies without UCB infusion are at a greater risk of developing GF/PGF and a greater risk for relapse. The patients who were negative for HLA antibodies still had better 1-year RFS, GRFS, and OS than those who were positive for HLA antibodies regardless of the UCB infusion. In the future, we will also expand the sample size to further validate our results.

## Data Availability

The raw data supporting the conclusions of this article will be made available by the authors, without undue reservation.
