# Supplementary material for: Umbilical cord blood stem cells as third-party adjuvant infusions in human leukocyte antigen antibody-positive patients undergoing haploidentical hematopoietic stem cell transplantation
Source: Front Immunol. 2024 Sep 27;15:1459699. doi: 10.3389/fimmu.2024.1459699 (PMC11466763; doi:10.3389/fimmu.2024.1459699)
Supplement: Supplementary file 1 [file Table1.docx]

**Supplementary Table S1 The characteristics of complications in all patients**

|  | No. | % |
| --- | --- | --- |
| Occurrence of aGVHD (n=90) |  |  |
| Yes | 49 | 54.4% |
| No | 41 | 45.6% |
| Grades of aGVHD |  |  |
| I | 17 | 34.7% |
| II | 20 | 40.8% |
| III-IV | 12 | 24.5% |
| aGVHD Organ Involvement |  |  |
| Skin | 23 | 46.9% |
| Liver | 1 | 2.1% |
| Gastrointestinal tract | 8 | 16.3% |
| Two or more organs involved | 17 | 34.7% |
| Occurrence of cGVHD (n=84) |  |  |
| Yes | 17 | 20.2% |
| No | 67 | 79.8% |
| cGVHD organ involvement |  |  |
| Lung（BOS） | 1 | 5.9% |
| Skin, joint, and connective tissue | 2 | 11.8% |
| Liver | 4 | 23.5% |
| Oral cavity | 4 | 23.5% |
| Two or more organs involved | 6 | 35.3% |
| CMV reactivation within 100 days |  |  |
| Yes | 43 | 47.8% |
| No | 47 | 52.2% |
| EBV reactivation within 100 days |  |  |
| Yes | 65 | 72.2% |
| No | 25 | 17.8% |
| Incidence of HC |  |  |
| Yes | 26 | 28.9% |
| No | 64 | 71.1% |
| Other infections |  |  |
| Pulmonary infection | 43 | 47.8% |
| Two or more other infections | 38 | 42.2% |
| Without others | 9 | 10.0% |

aGVHD, acute graft-versus-host disease; cGVHD, chronic graft-versus-host disease; BOS, bronchiolitis obliterans syndrome; CMV, cytomegalovirus; EBV, Epstein-Barr virus; HC, hemorrhagic cystitis.
